# Mental health and adherence to antiretroviral therapy among Mexican people living with HIV during the COVID-19 pandemic

**DOI:** 10.1186/s12981-023-00532-0

**Published:** 2023-06-07

**Authors:** Ester Gutiérrez-Velilla, Vania Barrientos-Casarrubias, María Gómez-Palacio Schjetnan, Lydia E. Perrusquia-Ortiz, Rosa Cruz-Maycott, Claudia Alvarado-de la Barrera, Santiago Ávila-Ríos, Nancy Patricia Caballero-Suárez

**Affiliations:** Centro de Investigación en Enfermedades Infecciosas (CIENI) del Instituto Nacional de Enfermedades Respiratorias (INER), Calzada de Tlalpan 4502, Belisario Domínguez Secc 16, Tlalpan, 14080 Ciudad de México, México

**Keywords:** HIV/AIDS, Mental health, ART adherence, COVID-19 pandemic

## Abstract

**Background:**

The mental health and medical follow-up of people living with HIV (PLWH) have been disrupted by the COVID-19 pandemic. The objectives of this study were to assess anxiety, depression and substance use in Mexican PLWH during the pandemic; to explore the association of these symptoms with adherence to antiretroviral therapy (ART), and to compare patients with and without vulnerability factors (low socioeconomic level, previous psychological and/or psychiatric treatment).

**Methods:**

We studied 1259 participants in a cross-sectional study, PLWH receiving care at the HIV clinic in Mexico City were contacted by telephone and invited to participate in the study. We included PLWH were receiving ART; answered a structured interview on sociodemographic data and adherence to ART; and completed the psychological instruments to assess depressive and anxiety symptoms and substance use risk. Data collection was performed from June 2020 to October 2021.

**Results:**

84.7% were men, 8% had inadequate ART adherence, 11% had moderate-severe symptoms of depression, and 13% had moderate-severe symptoms of anxiety. Adherence was related to psychological symptoms (p < 0.001). Vulnerable patients were more likely to be women, with low educational level and unemployed (p < 0.001).

**Conclusions:**

It is important to address mental health of PLWH during the COVID-19 pandemic, with special attention to the most vulnerable individuals. Future studies are needed to understand the relationship between mental health and ART adherence.

## Background

The COVID-19 pandemic has imposed significant burden of disease worldwide, as more than 500 million cases and more than 6 million deaths have been recorded worldwide [1]. In Mexico, more than 6 million cases and nearly 300.000 deaths had been recorded by July 2022 [2]. Safety measures have included isolation, use of facemasks, social distancing, and confinement. Paradoxically, these measures have promoted health mental issues in the population [3, 4]. Anxiety, depression, post-traumatic stress symptoms, sleep problems, irrational anger, and even suicidal behavior have been reported as consequences of the pandemic [5–7]. Some factors associated with mental health problems during the COVID-19 pandemic included comorbid physical and mental health problems, coping styles, stigma, psychosocial support, confidence in health services, risk of contracting COVID-19 or perceived likelihood of survival [5].

People living with HIV (PLWH) have been particularly affected by the COVID-19 pandemic due to the limited access to medical care, prevention measures, and treatment [8, 9]. During the pandemic, clinical follow-up could not be face-to-face in many cases [10] and health personnel have been overwhelmed with COVID-19 patients [11]. Also, economic problems have led to unemployment and increased poverty, making it difficult for vulnerable patients to transport themselves to collect their medications [12–14].

Similarly, the approach to mental health problems has been difficult during the pandemic due to the increased workload of health professionals [15, 16], but also due to the difficulty of providing face-to-face care [9, 17] as well as the economic and employment problems caused by the pandemic [12]. For the aforementioned reasons, alternative strategies of providing mental health care have included the use of technologies like telephone calls or video calls to evaluate and treat patients remotely [18–21]. In this sense, it has been demonstrated that these type of interventions improve outreach, allow to be performed remotely, with greater comfort and flexibility, and are also effective for psychological assessment and treatment [22, 23].

In these context of the pandemic, it became particularly important to closely monitor the development of mental health problems in vulnerable populations with preexisting high prevalence of these symptoms. PLWH are more likely to present anxiety and depressive symptoms than the general population [24, 25]. This is derived from circumstances surrounding their condition such as stigma and discrimination [26–28]. Comorbid psychiatric disorders can affect the quality of life of PLWH by increasing the likelihood of hopelessness, lack of interest in well-being, substance use, and risk behaviors [29]. In addition, these problems have been closely related to inadequate adherence to antiretroviral therapy (ART) and loss of clinical follow-up [30–33], which can lead to poorer health, progression to AIDS, development of resistance to ART and increased transmission of the virus [34–36]. Therefore, it is imperative to evaluate and address mental health problems in this population, especially in the most vulnerable individuals [37], to allow their adequate follow-up and adherence to ART. Therefore, the objectives of this study were to assess anxiety, depression and substance use in Mexican PLWH during the pandemic; to explore the association of these symptoms with adherence to ART, and to compare patients with and without vulnerability to mental health problems (low socioeconomic level, previous psychological and/or psychiatric treatment).

## Methods

### Setting and participants

The study was conducted in an adult HIV clinic at the National Institute of Respiratory Diseases in Mexico City. This hospital is the largest third-level national referral center for COVID-19 in Mexico. From early March 2020 the institution was repurposed for the treatment of patients with COVID-19 exclusively. The face-to-face care for HIV patients was canceled, medical appointments were provided through telemedicine (phone calls) and an organized system of appointments was implemented for ART refill and laboratory tests.

All patients receiving care at the HIV clinic (n = 1455) were contacted by telephone and invited to participate in the study. We included all patients who had a diagnosis of HIV; were receiving ART; answered a structured interview on sociodemographic data and adherence to ART; and completed the psychological instruments to assess depressive and anxiety symptoms and substance use risk. The duration of the evaluation phone call was between 20 and 40 min. Data collection was performed from June 2020 to October 2021.

The group of participants with vulnerability to mental health problems was compared vs. the group of participants without vulnerability. Participants with vulnerability were those who had psychological treatment at the institution during the 12 months prior to the pandemic, psychiatric treatment at the institution during the 12 months prior to the pandemic or those who had the lowest socioeconomic level. Patients in this group could meet one, two or three vulnerability criteria. Patients without vulnerability were those who did not meet any of these criteria. We established vulnerability criteria ad hoc for this study considering the association between poverty and mental health problems [38, 39] and due to the greater probability that PLWH has mental health problems especially if they had previous diagnosis [24, 25].

### Design

Cross-sectional study which evaluated mental health and adherence variables in a group of Mexican people living with HIV by telephone. The STROBE (Strengthening the Reporting of Observational Studies in Epidemiology) guidelines were followed when reporting the findings of the study.

### Procedure

PLWH were contacted via telephone by five trained health psychologists inviting them to participate in the study. Priority was given to calling patients who met any of the vulnerability criteria described above. Those giving verbal consent to participate answered a structured interview, previously reviewed and discussed by psychologists, to collect sociodemographic information and data about the number of ART doses taken in the last thirty days. In addition, the General Anxiety Disorder Scale (GAD-7) [40], the Patient Health Questionnaire (PHQ-9) [41, 42] and the Alcohol, Smoking, and Substance Involvement Screening Test (ASSIST) [43] were applied. Psychologists gave feedback to the participants, according to their anxiety, depression, and substance use scores. Participants with inadequate ART adherence, with moderate or severe levels of anxiety or depression, and those who obtained moderate or high risk of substance use were invited to receive psychological intervention via telephone. In this sense, users participated voluntarily and without receiving compensation beyond knowing their results and receiving attention if they needed it.

### Measures

Sociodemographic data were collected through a structured questionnaire including age, gender, civil status, educational level, occupation, sexual orientation, and city of residence. We searched the socioeconomic level assigned by the social work department of the institution, and data about previous psychological or psychiatric treatment and last viral load count available were obtained from clinical records.

The percentage of ART adherence on the last thirty days, based on a patient's self-report of missed doses, was used as an indirect method proposed by the Pan-American Health Organization to assess ART adherence [44], this taking into account that it is a simple measurement that has been shown to have the same biases as other types of more complex measurements [45]. The percentage was calculated by subtracting the number of missed doses from the number of doses prescribed in the last thirty days, divided by the number of doses prescribed multiplied by 100. A percentage ≤ 95% was considered inadequate. Also, questions about doses taken after hours and incomplete doses were asked, in order to later provide an adequate intervention.

Anxiety symptoms were measured by using the culturally-adapted Spanish version of GAD-7 [40] which was validated in Mexican population living with HIV. The GAD-7 is a self-report scale based on the Diagnostic and Statistical Manual of Mental Disorders (DSM-IV) criteria for generalized anxiety disorder, including seven items scored from “0” (not at all) to “3” (nearly every day). Scale scores ranged from 0 to 21 and cut-off scores were defined as 0–4 (no anxiety symptoms); 5–9 (mild anxiety symptoms); 10–14 (moderate anxiety symptoms); and 15–21 (severe anxiety symptoms).

Depression symptoms were assessed by using the culturally-adapted Spanish version of the PHQ-9 [41, 42] which was validated in Mexican population living with HIV. This is a self-applicable scale including 9 items based on the diagnostic criteria of the DSM-IV for depressive disorder. Answers ranged from “0” (not at all) to “3” (nearly every day). Scale scores ranged from 0 to 27. Cut-off scores were defined as 0–4 (no depression symptoms); 5–9 (mild depression symptoms); 10–14 (moderate depression symptoms); 15–19 (moderately severe depression symptoms); and 20–27 (severe depression symptoms).

Substance use risk was evaluated with the Spanish version of ASSIST V3.1, which is an 8-item questionnaire designed to be administered by a health worker to a patient, developed to be culturally neutral and useable across a variety of cultures and populations for the screening of risk of use of the following substances: tobacco products, alcohol, cannabis, cocaine, amphetamine-type stimulants, sedatives and sleeping pills (benzodiazepines), hallucinogens, inhalants, opioids, and other drugs. The ASSIST determines a risk score for each substance. The score obtained for each substance falls into a ‘lower’, ‘moderate’, or ‘high’ risk category. The ASSIST obtains information from individuals about lifetime use of substances, and use of substances and associated problems over the last three months [43].

### Data analysis

Sociodemographic variables, percentage of ART adherence, the severity level of anxiety and depression symptoms, and the risk of substance use were described by frequencies, percentages, means, and standard deviations (SD). The t-Student test and chi-square test were used to compare sociodemographic variables, ART adherence, and mental health variables between individuals with vulnerability vs. those without vulnerability. To calculate the sample size, the GPower statistical program was used [46], because the means of two groups were to be compared, and the ratio between the groups was 3–1, to find a mean effect size, with a probability of error of 0.05 a sample of at least 280 participants is needed, 70 participants in the vulnerability group and 210 participants in the non-vulnerability group. We used Pearson's correlation coefficients to explore possible associations between ART adherence and mental health variables between both groups (with and without vulnerability). Finally, a multiple linear regression analysis was performed on the outcome variable adherence to ART. Vulnerability, mental health problems (anxiety, depression, and substance use) and sociodemographic variables were introduced into the model to control for their effects. Collinearity diagnostics were performed with the tolerance and VIF indices, expecting them to be less than 2. Analyses were performed with IBM SPSS Statistics version 25 software and values were considered significant if p < 0.05.

### Ethical considerations

The Institutional Research Board of the Instituto Nacional de Enfermedades Respiratorias (INER) reviewed and approved the study (Protocol Number: C31-20). Given restrictions imposed by the COVID-19 pandemic, and the minimal risk of the study, and as allowed by Council of International Organizations of Medical Sciences under these circumstances [47], the Institutional Research Board authorized the participants were invited by telephone and to give their verbal informed consent to participate in this study after having read the informed consent form to them and sent it by mail if they requested it.

## Results

### Sociodemographic and clinical data of participants

Of the 1,455 patients receiving care at our clinic, 1259 were included in the study. A total of 196 patients were not included: 46 did not agree to participate; 12 could not answer the interview due to medical conditions such as cognitive impairment or medical devices that prevented them from speaking (e.g. tracheostomy); 112 could not be located in attempted phone calls; and 26 were being treated at another clinic. The mean age was 42 years (SD = 10.11). Most participants were male (84.7%), single (69.6%), and residents of Mexico City (68.3%). Seventy percent of the participants were employed (either formally or informally), and most of them identified themselves as homosexual (61.8%). Concerning vulnerability criteria, 9% had required psychological treatment in the 12 months prior to the pandemic, and 7% had psychiatric treatment. Slightly more than 20% of the participants met at least one vulnerability criteria (previous psychiatric or psychological treatment or having the lowest socioeconomic level). Also, according to the last collected viral load count, at least 94% of the participants had undetectable viral load and only around 6% had detectable viral loads (the mean log viral load was 2.3 copies/ml, S.D. = 0.97). Sociodemographic characteristics of study participants and comparison between groups with and without vulnerability are shown in Table 1.

**Table 1:** Sociodemographic characteristics of study participants and comparison between groups with and without vulnerability

	Total n = 1259	Vulnerability***		p-value
		No n = 983	Yes n = 276	
Age				
Mean	42.78	42.77	42.79	t = – 0.022
SD	10.11	9.96	10.64	p = 0.98
Range	20–74	20–74	22–68	
Gender % (n)				
Male	84.7 (1067)	87.4 (859)	75.4 (208)	x^2^ = 28.09
Female	13.3 (167)	10.6 (104)	22.8 (63)	p < 0.001
Transgender	2.0 (25)	2.0 (20)	1.8 (5)	
Place of residence % (n)				
Mexico City	68.3 (860)	69.3 (681)	64.9 (179)	x^2^ = 4.21
State of Mexico	21.3 (268)	20.0 (197)	25.7 (71)	p = 0.121
Other	10.4 (131)	10.7 (105)	9.4 (26)	
Marital status % (n)				
Single	69.6 (876)	71.8 (706)	61.6 (170)	
Married	11.8 (148)	11.5 (113)	12.7 (35)	x^2^ = 20.31
In common law	11.8 (148)	11.3 (111)	13.4 (37)	p < 0.001
Separated	4.1 (52)	3.4 (33)	6.5 (18)	
Widowed	2.9 (36)	2.0 (20)	5.8 (16)	
Educational level % (n)				
Unschooled	2.7 (34)	2.0 (20)	5.1 (14)	
Basic education	32.5 (409)	28.2 (277)	47.8 (132)	x^2^ = 68.85
High school education	32.6 (411)	34.9 (343)	24.7 (68)	p < 0.001
Superior education	32.1(405)	34.9 (343)	22.5 (62)	
Employed % (n)				
Unemployed	22.1 (278)	18.9 (186)	33.3 (92)	
Informal employment	35.4 (446)	36.5 (359)	31.5 (87)	x^2^=61.68
Formal employment	35.2 (443)	38.8 (381)	22.5 (62)	p<0.001
Housewife/husband	4.6 (58)	3.1 (30)	10.1 (28)	
Student	2.2 (28)	2.2 (22)	2.2 (6)	
Retired	0.5 (6)	0.5 (5)	0.4 (1)	
Sexual orientation % (n)				
Heterosexual	31.3 (394)	27.6 (270)	45.8 (124)	
Homosexual	61.8 (778)	65.5 (641)	50.6 (137)	
Bisexual	4.8 (61)	5.5 (54)	2.6 (7)	x^2^ = 33.64
Other	1.3 (16)	1.3 (13)	1.1 (3)	p < 0.001
Missing data	0.8 (10)	27.6 (270)	45.8 (124)	
Socioeconomic level* % (n)				
1x	11.4 (144)			
1	37.2 (468)			
2	40.1 (505)	42.77	42.79	
3	9.2 (116)	9.96	10.64	t = -0.022
4	1.7 (22)	20–74	22–68	p = 0.982
5	0.2 (3)			
6	0.1 (1)			
Previous psychological treatment ** % (n)				
No	90.9 (1145)			
Yes	9.1 (114)			
Previous psychiatric treatment ** % (n)				
No	92.9 (1170)			
Yes	7.1 (89)			
Vulnerability criteria*** % (n)				
0	78.1 (983)			
1	16.9 (213)			
2	4.4 (56)			
3	0.6 (7)			

^SD^^: S^^tandard deviation^

*Social work staff assigns this socioeconomic classification to each patient treated at the clinic to determine the fees for clinical services and several indicators are considered: occupation, monthly income, type of housing, number of people patients live with, number of economic providers and dependents. 1x is the lowest socioeconomic level (exempt from payment), while 6 is the highest

**Participants had psychological and/or psychiatric treatment in the 12 months previous to the health contingence

***Vulnerability criteria established were: (1) socioeconomic level 1x, (2) previous psychological treatment, (3) previous psychiatric treatment. Participants with at least 1 vulnerability criteria were prioritized to be contacted

When comparing sociodemographic characteristics between people with (21.9% n = 276) and without (78.1% n = 983) vulnerability criteria, significant differences were found in gender, marital status, educational level, occupation, and sexual orientation. Specifically, in the group with vulnerability, there were more women (p < 0.001), more divorced and widowed people (p < 0.001), more people with low educational levels (p < 0.001), more unemployed (p < 0.001), and more heterosexuals (p < 0.001). Table 1 shows the comparison of sociodemographic variables between people with and without vulnerability.

### Psychological variables and ART adherence

Regarding psychological and adherence variables, 7.7% of participants had inadequate adherence to ART (< 95%), 11.5% had moderate to severe symptoms of depression, 13% had moderate to severe symptoms of anxiety; and 8.3% had comorbidity of anxiety and depression symptoms. Also, 21% had moderate to high risk of tobacco use, 3% had moderate to high risk of alcohol or cannabis use, and 1% had moderate to high risk of cocaine use. When comparing people with and without vulnerability, statistically significant differences were found in depressive and anxious symptomatology and risk of cocaine use. Specifically, in the group with vulnerability, scores on depression and anxiety scale were higher (p < 0.001) and a higher proportion of participants presented a moderate risk of cocaine use (p < 0.001). Table 2 shows the psychological variables in both groups and the total population, as well as their comparison.

**Table 2:** Comparison of psychological variables between groups with and without vulnerability

		Total n = 1259	Vulnerability			p-value
			No n = 983		Yes n = 276	
Percentage with suboptimal ART Adherence* Mean SD Range		98.71 5.276 0–100	98.67 5.79 0–100		98.86 2.75 80–100	t = -0.758 p = 0.601
Percentage of ART Adherence % (n) 96%-100% 80%-95% 0%-79%		92.3 (1162) 7.0 (88) 0.5 (6)	92.6 (910) 6.5 (64) 0.6 (6)		91.3 (252) 8.7 (24) 0	x^2^ = 3.19 p = 0.203
PHQ-9 total score Mean SD Range		4.22 4.65 0–26	3.77 4.26 0–24		5.83 5.55 0–26	t = -6.58 p < 0.001
PHQ-9 level of symptomatology % (n) None (0–4 points) Mild depression (5–9 points) Moderate depression (10–14 points) Moderately severe depression (15- 19 points) Severe depression (20–27 points)		67.4 (848) 21.1 (266) 6.8 (86) 3.2 (40) 1.5 (19)	71.9 (707) 19.2 (189) 5.2 (51) 2.5 (25) 1.1 (11)		51.1 (141) 27.9 (77) 12.7 (35) 5.4 (15) 2.9 (8)	x^2^ = 49.46 p < 0.001
GAD-7 total score Mean SD Range		4.61 4.19 0–21	4.22 3.98 0–21		6.00 4.62 0–20	t = -6.30 p < 0.001
GAD-7 level of symptomatology % (n) None (0–4 points) Mild depression (5–9 points) Moderate depression (10-14 points) Severe depression (15–21 points)		59.7 (751) 27.4 (345) 9.5 (119) 3.5 (44)	63.9 (628) 25.6 (252) 7.2 (71) 3.3 (32)		44.6 (123) 33.7 (93) 17.4 (48) 4.3 (12)	x^2^ = 42.90 p < 0.001
ASSIST risk of tobacco use % (n) Low Moderate High		79.1 (996) 20.7 (260) 0.2 (3)	79.5 (781) 20.2 (199) 0.3 (3)		77.9 (215) 22.1 (61) 0	x^2^ = 1.27 p = 0.530
ASSIST risk of alcohol use % (n) Low Moderate High		96.6 (1216) 2.9 (37) 0.5 (6)	96.9 (953) 2.6 (26) 0.4 (4)		95.3 (263) 4.0 (11) 0.7 (2)	x^2^ = 1.84 p = 0.399
ASSIST risk of cannabis use % (n) Low Moderate High		96.7 (1217) 3.3 (42) 0	96.8 (952) 3.2 (31) 0		96.0 (265) 4.0 (11) 0	x^2^ = 0.462 p = 0.496
ASSIST risk of cocaine use % (n) Low Moderate High		99.0 (1246) 1.0 (12) 0.1 (1)	99.4 (977) 0.5 (5) 0.1 (1)		97.5 (269) 2.5 (7) 0	x^2^ = 9.66 p = 0.008

^SD^^: S^^tandard deviation. t^^: t^^−^^Student test, x2^^: C^^hi^^−^^square test^

^*The percentage was calculated by subtracting the number of missed doses from the number of doses prescribed in the last thirty days, divided by the number of doses prescribed multiplied by 100, a percentage ≤95% was considered inadequate^

Adherence had a low but significant correlation with all psychological variables. Anxiety and depression symptoms were highly correlated (r = 0.781, p < 0.001), and these had a low but significant correlation with the risk of use of all substances. Finally, the risk of using one substance was related to the risk of using other substances (Table 3).

**Table 3:** Correlation of ART adherence with psychological variables and risk of substance use

	PHQ-9 total score	GAD-7 total score	Tobacco assist score	Alcohol assist score	Cannabis assist score	Cocaine assist score
% ART adherence Total Vulnerability Without vulnerability	− 0.138^**^ − 0.253^**^ − 0.135^**^	− 0.119^**^ − 0.258^**^ − 0.110^**^	− 0.061^*^ − 0.035 − 0.068^**^	− 0.106^**^ − 0.141^*^ − 0.107^**^	− 0.024 − 0.140^*^ − 0.004	− 0.116^**^ − 0.086^**^ − 0.119^**^
PHQ-9 total score Total Vulnerability Without vulnerability	1	0.781^**^ 0.746^**^ 0.787^**^	0.174^**^ 0.202^**^ 0.160^**^	0.209^**^ 0.190^**^ 0.224^**^	0.104^**^ 0.058 0.122^**^	0.149^**^ 0.030 0.186^**^
GAD-7 total score Total Vulnerability Without vulnerability	0.781^**^	1	0.156^**^ 0.138^*^ 0.159^**^	0.205^**^ 0.186^**^ 0.219^**^	0.165^**^ 0.085 0.201^**^	0.139^**^ 0.053 0.162^**^
Tobacco ASSIST score Total Vulnerability Without vulnerability	0.174^**^	0.156^**^	1	0.254^**^ 0.142^*^ 0.296^**^	0.147^**^ 0.118 0.161^**^	0.202^**^ 0.194^**^ 0.207^**^
Alcohol ASSIST score Total Vulnerability Without vulnerability	0.209^**^	0.205^**^	0.254^**^	1	0.115^**^ 0.143^*^ 0.103^**^	0.267^**^ 0.045 0.326^**^
Cannabis ASSIST score Total Vulnerability Without vulnerability	0.104^**^	0.165^**^	0.147^**^	0.115^**^	1	0.234^**^ − 0.034 0.327^**^

^Pearson’s correlation coefficients, **the correlation is significant at the 0.01 level (bilateral), *the correlation is significant at the 0.05 level (bilateral)^

Multiple regression analysis on ART adherence was significant F(_14,1240)_ = 4.786, p < 0.001, and although the adjusted R^2^ was very small (0.041), some variables were found to predict adherence to ART. Specifically, higher educational levels, both high school (B = 1.367, 95 CI 0.632 to 2.103, p = 0.001), and university (B = 1.474, 95 CI 0.727 to 2.221, p = . 001) increased adherence to ART; on the other hand, higher scores on the depression instrument (B = -0.109, 95 CI − 0.209 to − 0.009, p = 0.032) and higher risk of alcohol consumption (B = -0.81, 95 CI − 0.159 to − 0.003, p = 0.042) were related to reduced reported adherence to ART. Table 4 shows the coefficients, confidence intervals and p-values of all the variables in the model.

**Table 4:** Multiple regression model of ART adherence (n = 1259)

	B	SD	95%	CI	t		p
Vulnerability^a^	0.735	0.375	0.000	1.471	1.961		0.050
Gender^b^	0.469	0.440	− 0.395	1.332	1.065		0.287
Age	0.021	0.015	− 0.008	0.050	1.447		0.148
Basic education							
High school	1.367	0.375	0.632	2.103	3.648		0.000**
University	1.474	0.381	0.727	2.221	3.872		0.000**
Employment^a^	− 0.374	0.335	− 1.032	0.284	− 1.115		0.265
Stable partner^a^	0.352	0.296	− 0.229	0.934	1.189		0.235
Previous lost to follow up^a^	− 0.742	0.437	− 1.600	0.116	− 1.697		0.090
Score on anxiety symptoms	− 0.049	0.057	− 0.160	0.063	− 0.857		0.391
Score on depression symptoms	− 0.109	0.051	− 0.209	00.009	− 2.147		0.032*
Risk of tobacco	− 0.008	0.031	− 0.069	0.053	− 0.265		0.791
Risk of alcohol	− 0.081	0.040	− 0.159	− 0.003	− 2.031		0.042*
Risk of marihuana	0.024	0.090	− 0.152	0.200	0.271		0.786
Risk of cocaine	− 2.073	1.376	− 4.774	0.627	− 1.506		0.132

^SD=standard deviation; CI=Confidence interval; a:0=No, 1=Yes; b:0=Male, 1=Female; *p<.05; **p<.001^

## Discussion

The main objective of this study was to describe mental health problems in Mexican people living with HIV during the COVID-19 pandemic. We found that 13% of the population studied had moderate-severe symptoms of anxiety, and 11% had moderate-severe symptoms of depression. These percentages may seem low, compared to other studies conducted in PLWH during the same period. In a cohort in New York, anxious symptomatology was reported in 43% of the participants and depressive symptomatology in 45%, although measurements were performed with the PHQ-2 and GAD-2 instruments, which may be less specific [48]. In other studies, conducted with PLWH in the United States, around 30% of participants presented moderate-severe symptoms of depression [49, 50]. A possible explanation for the lower levels of mental health problems found in our study could be that patients attending our institution are well-controlled, most with undetectable viral load. Therefore, it is a population that have high levels of adherence and are generally in good health, so they may perceive fewer risks and may not suffer as much anxiety as other uncontrolled populations. The risk of substance use, except for tobacco, was also lower in our population, compared to other studies conducted during the pandemic where up to 13% of initiation and increase in substance use has been reported [49].

Seven percent of the population studied reported inadequate adherence (< 95%) to ART, which was similar to other studies conducted in Latin America, reporting values of 5–12% [51, 52], and this was found to be related to educational level, the level of depression and alcohol consumption. Monitoring self-reported adherence to ART during the COVID-19 pandemic is especially relevant, as other studies have reported up to 14% of PLWH considered that the pandemic had decreased their adherence [53]. The reported causes of decreased ART adherence during the pandemic include the lack of life structure derived from the mandatory stay-at-home and the limited access to medication [54]. One possible explanation for the relatively high reported adherence in this study could be the belief that ART could work against COVID-19 infection, so many people with HIV became very adherent [55].

In our study, people with greater vulnerability to mental health problems (previous psychological/psychiatric treatment and low educational level) were more likely to be women, unemployed, and with low educational levels. This is consistent with the fact that women, during the pandemic, have reported more mental health problems and difficulties in maintaining adequate ART adherence during the pandemic [52, 56, 57]. Besides, being female has been recognized as a psychosocial vulnerability factor, both in the general population [58] and in PLWH [39, 59]. On the other hand, patients with previous vulnerability in this study presented greater problems of anxiety, depression, and substance use, consistent with other studies reporting a greater likelihood of emotional symptoms in patients with a history of mood disorder [48, 55]. With this in mind, it is important to monitor closely those individuals who are more likely to have mental health problems, considering the impact that these diagnoses can have on ART adherence and other risk behaviors, such as attendance at appointments, and their close relationship with health outcomes [30, 34, 35].

Throughout the study period, Mexico experienced different COVID waves that increased the number of cases and deaths, with 200,000 confirmed cases of COVID-19 at the beginning of the study and more than 3 million confirmed cases by the end of the study. This, combined with the implementation of isolation and social distancing measures, could have affected the results of this study across the different evaluation time points [60]. However, to account for this, the levels of mental health and adherence to ART were compared depending on the time of evaluation and we found that there were no significant differences between the distinct waves.

Although the study provides important results, there are some limitations that should be taken into account. First, the definition of vulnerability may include biases, as there may be vulnerable individuals who were not assessed or treated within the established time period. This could be due to stigma about receiving care, lack of access to health services, or even poor recording of health information. For this reason, caution should be taken when generalizing the results and keep in mind that these biases could be present. On the other hand, ART adherence information was obtained by self-report. Although this is a common way to measure adherence behaviors, it may represent a recall bias or social desirability bias in patients interviewed, therefore, this possible bias, which has also been reported in other more complex measurements such as psychometric scales, must be taken into account [45]. Related to this, the time that the participants had been on antiretroviral treatment was not measured, it is a variable important to take into account, since this may affect adherence, it will not be the same if a patient is starting to use antiretroviral treatment and is adapting to a new medication routine, or if it has been under treatment for years. For this reason, we consider it important to carry out future studies that include the measurement of time under antiretroviral treatment. Another potential limitation of the study was the use of telephone interviews, as this method could lead to loss of non-verbal data, loss of contextual data or distortion of verbal data, in addition to the fact that people without access to a telephone could have been left out of the study, for that reason it is proposed that in future studies, an exhaustive search for people with limited resources and who cannot access communication channels such as the telephone, was made. Finally, the study was cross-sectional and performed in only one center, so it would be important to perform multi-center longitudinal studies to assess the impact of the pandemic on PLWH mental health and ART adherence in the long term and with higher representativeness.

## Conclusions

This study found that people with previous psychological vulnerability were more likely to present current mental health problems, such as anxiety, depression and substance use. In addition, adherence to antiretroviral treatment was found to be related to mental health problems as depression and alcohol consumption, and with educational level. Considering the difficulties in maintaining ART adherence and the increased risk of developing mental health problems during the pandemic, it is essential to monitor the mental health of PLWH, especially of those with a history of mental health problems. It is important to pay special attention to women, people with scarce resources and educational level, since they are at greater risk of presenting psychological vulnerability. Prevention strategies and effective psychological interventions to address these mental health symptoms in PLWH should be designed.

## Data Availability

The current article includes the complete raw data set collected in the study including the participants' data set. The data file was uploaded to the Figshare repository https://doi.org/10.6084/m9.figshare.20383728.
